
JOURNAL INFORMATION
==============================
NLM Title Abbreviation: Eur J Gen Pract
Journal ID: Eur J Gen Pract
NLM Journal ID: 9513566

The European Journal of General Practice

ISSN: 1381-4788
EISSN: 1751-1402
Publisher: Taylor & Francis

ARTICLE INFORMATION
==============================
PMCID: PMC12392425
DOI: 10.1080/13814788.2025.2542288
Article ID: 2542288
Article version: 1
Article version: Version of Record
Subjects: Abstract, Abstracts

100th EGPRN Meeting, 8–11 May 2025, Gothenburg – Sweden

Abstracts

Kaya Bezgin Mine
Electronic publication date: 2025 Aug 27
Publication date: 2025
Volume: 31
Issue: 1
Electronic Location ID: 2542288
Copyright: © 2025 The Author(s). Published by Informa UK Limited, trading as Taylor & Francis Group.
Copyright year: 2025
Copyright holder: The Author(s)
License: This is an Open Access article distributed under the terms of the Creative Commons Attribution License (http://creativecommons.org/licenses/by/4.0/), which permits unrestricted use, distribution, and reproduction in any medium, provided the original work is properly cited. The terms on which this article has been published allow the posting of the Accepted Manuscript in a repository by the author(s) or with their consent.
License URL: https://creativecommons.org/licenses/by/4.0/

Erratum in: Correction 31 1 8 9 2025 2557121 The European Journal of General Practice 10.1080/13814788.2025.2557121 PMC12418793 40919607
Figure count: 0
Table count: 0
Page count: 8

==============================


Telehealth: Its benefits, quality, and safety

Subjects: Abstract, International Keynote Lecture

Telehealth: Access to acute primary care

Huibers Linda
Department of Public Health, Institute of General Medical Practice, Aarhus University , Aarhus , Denmark
CONTACT Linda Huibers huibers@ph.au.dk

Subjects: Abstract, National Keynote Lecture

AI in Swedish primary care – a GP’s ultimate bucket list

Nymberg Veronica Milos
Center for Primary Care Research Faculty of Medicine Department of Clinical Sciences, Malmö Lund University , Lund , Sweden
CONTACT Veronica Milos Nymberg Veronica.milos_nymberg@med.lu.se

Subjects: Abstract, Poster Prize Winner

Effect of ‘diaphragmatic breathing exercises’ on functional constipation, anxiety and depression

Ölke Çigdem
Unalan Pemra C.
Uzuner Arzu
Kocabas Pınar
Marmara University, School of Medicine, Department of Family Medicine , Istanbul , Türkiye
CONTACT Pınar Kocabas pcunalan@gmail.com
Keywords: Functional constipation, constipation, diaphragmatic breathing, belly breathing, anxiety, depression

Subjects: Abstract, Theme Papers

The obscured face in video consultations: A qualitative analysis of patient experiences

Hvidt Elisabeth Assing
Kofod Frida Greek
Van Den Heuvel Johannes
Scheffmann-Petersen Michael
Department of Public Health, Research Unit of General Practice , Odense , Denmark
CONTACT Elisabeth Assing Hvidt ehvidt@health.sdu.dk
Keywords: Video consultations, general practice, phenomenology, levinas, buber, ethical telecare

Subjects: Abstract, Theme Papers

Value appraisal of digital triage platforms

Hendriksen Ingrid
De Lange Wendela
Doorn Sander Van
Van Vugt Vincent
NHG , Utrecht , Netherlands
CONTACT Ingrid Hendriksen i.hendriksen@nhg.org
Keywords: digital triage, value appraisal, scoping review

Subjects: Abstract, Theme Papers

The effect of medical information being transported via digital tools on the level of knowledge of general medical issues and breast cancer among breast cancer patients

Azuri Joseph
Abo Chen
Tel Aviv University and Maccabi Helathcare Services , Tel Aviv , Israel
CONTACT Joseph Azuri azurij@actcom.co.il

Subjects: Abstract, Theme Papers

Determinants of the use of teleconsultations by doctors and depressed patients in their follow-up in France

Mercier Alain
Université Paris 13 , ERMONT , France
CONTACT Alain Mercier alain.mercier@univ-paris13.fr
Keywords: Depressed patients, telehealth

Subjects: Abstract, Theme Papers

After-hours telemedicine paediatric medical consultation centre – does it meet medical needs?

Vinker Shlomo
Shuster Michal
Elad Aviva
Cohen Avivit Golan
Family Meidcine, Leumit Health Services , Ashdod , Israel
CONTACT Shlomo Vinker vinker01@gmail.com
Keywords: Telemedicine, paediatrics, healthcare services, overtreatment

Subjects: Abstract, Theme Papers

Primary care in the age of telemedicine: Diagnoses and physician time across visit modalities

Schonmann Yochai
Aizenbud Lilach
Clalit Health Services , Tel Aviv , Israel
CONTACT Yochai Schonmann yochais@gmail.com
Keywords: primary care, visit duration, diagnoses, time allocation, telemedicine, electronic health records, reasons for encounter

Subjects: Abstract, Freestanding Papers

Inter-contact intervals for temporal discrimination of acute from chronic care in general practice

Hauswaldt Johannes
Hummers Eva
Himmel Wolfgang
Institute of General Practice, University Medical Center Göttingen , Göttingen , Germany
CONTACT Johannes Hauswaldt johannes.hauswaldt@med.unigoettingen.de
Keywords: Continuity of care, acute care, chronic condition, multimorbidity, frequent attendance

Subjects: Abstract, Freestanding Papers

Letters to general practice: Exploring female GPs’ experiences and expectations of a sustainable career in general practice

Keenan Ivana
Barrett Aileen
Brown Megan
Collins Claire
Irish College of General Practitioners , Dublin , Ireland
CONTACT Ivana Keenan Ivana.Keenan@ICGP.IE
Keywords: Female GPs, career sustainability, general practice

Subjects: Abstract, Freestanding Papers

De-implementation of low-value care practices in primary care: Results from the DE-imFAR study on abandonment of low-value pharmacological prescription for cardiovascular disease primary prevention

Rogers Heather L.
Sanchez Alvaro
The De-Imfar Phase 2 Study Team
BioBizkaia Health Research Institute , Barakaldo , Spain
CONTACT Heather L. Rogers rogersheatherl@gmail.com
Keywords: Implementation science, preventive medicine, cardiovascular diseases

Subjects: Abstract, Freestanding Papers

Gender bias in assessing chest pain among general medical trainees and general practitioners in Western brittany, France

Clement Alice
Dany Antoine
Barais Marie
Departement Universitaire de Médecine Générale, Université de Bretagne Occidentale , Brest cedex 3 , France
CONTACT Marie Barais marie.barais@gmail.com
Keywords: Gender, chest pain, ımplicit bias, gender bias, general practice

Subjects: Abstract, Freestanding Papers

Socio-demographic factors of childhood vaccine hesitancy in Albania

Qatipi Ledia
Fico Albana
Biles Mandy
University of Medicine in Tirana , Tirana , Albania
CONTACT Ledia Qatipi qatipilediamd@gmail.com
Keywords: Socio-demographic factors, vaccine hesitancy, childhood vaccination, Albania

Subjects: Abstract, Freestanding Papers

Climate change and health ‘through primary care physicians’ eyes’: A qualitative study in Greece

Vasileiadou Despoina
Pagkozidis Ilias
Christina-Maria Kravvari
Makris Konstantinos C.
Tsimtsiou Zoi
MSc Social–Preventive Medicine and Quality in Healthcare, Laboratory of Hygiene, Social & Preventive Medicine and Medical Statistics, School of Medicine, Aristotle University of, Thessaloniki , Thessaloniki , Greece
CONTACT Ilias Pagkozidis pagoilias@gmail.com
Keywords: Climate change, health, health literacy, Primary Health Care, physicians, qualitative research, perceptions

Subjects: Abstract, Posters

Effectiveness of bariatric surgery versus lifestyle weight loss interventions in adolescents: A long-term study of weight management and nutrient levels

Reuveni Miri Mizrahi
Cohen Bar
Atias Dor
Yehoshua Ilan
Adler Limor
Israel Association of Family Physicians , Sde Yoav , Israel
CONTACT Limor Adler adler_l@mac.org.il
Keywords: Obesity, adolescents, bariatric surgery, lifestyle weight loss intervention

Subjects: Abstract, Posters

Career choices of medical graduates who completed a novel general practice-orientated pre-graduate curriculum in Switzerland

Corpataux Océane
Pasche Olivier
Rodondi Pierre-Yves
Podmore Clara
Institute of Family Medicine, University of Fribourg , Fribourg , Switzerland
CONTACT Océane Corpataux oceane.corpataux@unifr.ch

Subjects: Abstract, Posters

Patients’ perspectives on facilitators to deprescribing medications in older patients in Sweden – a questionnaire study

Parodi López Naldy
Thulesius Hans
Mannheimer Stina
Jungo Katharina T.
Weir Kristie R.
Rozsnyai Zsofia
Streit Sven
Lüthold Renata Vidonscky
Sahlgrenska University Hospital , Gothenburg , Sweden
CONTACT Naldy Parodi López naldy.parodi.lopez@gu.se
Keywords: Deprescribing, older patients, polypharmacy, primary care

Subjects: Abstract, Posters

Choosing Wisely: Low value care as described by Swedish General Practitioners

Svensson Staffan
Pétursson Hálfdán
Mossberg Karin
Zettermark Sofia
Thörneby Andreas
Johansson Minna
Sjögreen Jonas
Lindfors Oskar
Gyll David
Hultberg Josabeth
Håkansson Jan
Bergsjön Nötkärnan Health Centre , Gothenburg , Sweden
CONTACT Staffan Svensson staffan.svensson@pharm.gu.se
Keywords: Low value care, choosing wisely, general practice

