# Correction

**DOI:** 10.1080/13814788.2025.2557121

**Published:** 2025-09-08

**Article title:** 100th EGPRN Meeting, 8–11 May 2025, Gothenburg – Sweden

**Author:** Mine Kaya Bezgin

**Journal:** *European Journal of General Practice*

**Bibliometrics:** Volume 31, Number 01, 2542288

**DOI:** https://doi.org/10.1080/13814788.2025.2542288

The introduction of this article was not included in the initial publication of the manuscript; however, it has now been added. Please see the introduction below:

Telehealth: Its Benefits, Quality, and Safety

The theme “Telehealth: Its Benefits, Quality, and Safety” is timely in today’s healthcare environment, where digital innovations are rapidly transforming care delivery. The COVID-19 pandemic accelerated telehealth adoption, revealing both its potential and challenges in ensuring effective, accessible, and safe healthcare.

Key areas of focus for this conference included:

- Telehealth’s Benefits: Improving access to care, especially for vulnerable and remote populations.
- Quality in Virtual Care: Maintaining high standards of care through virtual platforms, supported by AI-driven diagnostics and personalized treatment.
- Safety in Telehealth: Ensuring the security of patient data and the reliability of AI-enhanced technologies.
- AI’s Impact: Exploring how AI shapes telehealth with predictive analytics and clinical decision support, while addressing ethical considerations.
- Telehealth for All: Reducing healthcare disparities through broader access and equitable digital solutions.
- Policy and Regulation: Adapting regulatory frameworks to ensure the safe and effective use of telehealth and AI.

This conference provided a crucial platform for healthcare professionals, researchers, and policymakers to exchange ideas and best practices, contributing to the global effort to deliver safe, high-quality, and accessible healthcare through telehealth and AI innovations.

